# Metal-enhanced fluorescence and excited state dynamics of carotenoids in thin polymer films

**DOI:** 10.1038/s41598-019-40446-4

**Published:** 2019-03-05

**Authors:** Jaebeom Lee, Junghyun Song, Daedu Lee, Yoonsoo Pang

**Affiliations:** 1 Department of Physics and Photon Science, Gwangju Institute of Science and Technology, 123 Cheomdangwagi-ro, Buk-gu, Gwangju, 61005 Republic of Korea; 2 Department of Chemistry, Gwangju Institute of Science and Technology, 123 Cheomdangwagi-ro, Buk-gu, Gwangju, 61005 Republic of Korea

## Abstract

Metal-enhanced fluorescence of carotenoids, all-*trans*-β-carotene and 8′-apo-β-carotene-8′-al dispersed in thin layers of polystyrene and polyethylene glycol were investigated by time-resolved fluorescence spectroscopy. The weak emission signals of carotenoids in polymer films were increased by 4–40 times in the presence of a silver island film and the emission lifetimes of both carotenoids were measured as significantly shortened. The energy transfer from the intermediate states of carotenoids to the silver islands and the subsequent surface plasmon coupled emission were proposed for the mechanisms of metal-enhanced fluorescence. The fluorescence enhancements of carotenoids in the polymer films were also investigated statistically over a wide area of the silver island films.

## Introduction

Carotenoids are accessory pigments in natural photosynthetic systems in plants and photosynthetic bacteria. Carotenoids are known to have many roles in photosynthesis including light harvesting^1–3^ and photoprotection^4,5^. Diverse roles of carotenoids in natural photosynthesis might be related to their specific electronic structures and dynamics, which can be generally described by three singlet states of the conjugated polyene backbone of carotenoids^6–8^.

Photoexcitation into a *bright* S_2_ excited state of carotenoids converts on ultrafast time scales (less than a few hundred of femtoseconds) to an intermediate *dark* S_1_ excited state, which subsequently converts to the ground state, S_0_. Although excited state dynamics of carotenoids have been extensively studied experimentally by time-resolved electronic and vibrational spectroscopy^9–16^ and theoretically by time-dependent density functional theory methods^17–20^, details of the excited state dynamics of carotenoids, for example, the existence of intermediate dark states between the S_2_ and S_1_ state and the electronic structure and dynamics in the hydrogen bonding environment of the photosynthetic proteins, are still in question^6^.

**Figure 1:**
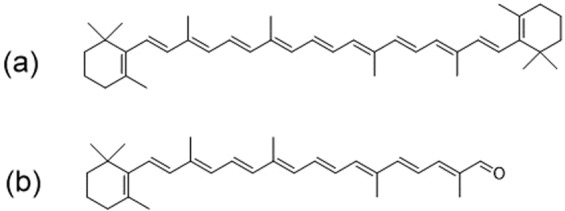
Molecular structure of (**a**) all-*trans*-β-carotene (carotene) and (**b**) 8′-apo-β-caroten-8′-al (carotenal).

All-*trans*-β-carotene (carotene) and 8′-apo-β-caroten-8′-al (carotenal) shown in Fig. 1 are examples of representative carotenoids often found in many plants and vegetables. The S_2_ and S_1_ energy levels of carotene are known as 20,840 and 14,500 cm^−1^, respectively^21,22^. The S_2_ energy level of carotenal, which is strongly dependent on the solvent polarity, is known as 20,100 (in hexane) and 18,400 cm^−1^ (in CS_2_), while the S_1_ energy level is known as 15,300 cm^−1^ ^23,24^. The excited-state lifetimes of both carotenoids are on the ultrashort time scales. The S_2_ lifetime of carotene was reported as 120–180 fs by Macpherson and Gillbro^21,25^, and slightly faster lifetimes of 95–120 fs were reported for carotenal by Mimuro *et al*.^26,27^. The S_1_ lifetimes of both carotenoids were known as 10–25 ps, which is strongly dependent on the energy level of the S_1_ state and called the energy gap law^28^. Both carotenoids show weak emissions from the S_2_ and S_1_ states^27,29–33^ with very small quantum yields of 1.1–1.7 × 10^−4^ for carotene^25,32^ and 1.8–2.4 × 10^−5^ for carotenal^27^.

Existence of intermediate states between the S_2_ and S_1_ have been reported for the excited state dynamics of both carotenoids^6,9,10,34–36^. A branching dynamics into a long-lived excited state (>1 ns) which may originate from a structural conformer in the S_1_ potential surface was also reported for carotenal^9,10^. Emissions from the intermediate states between the S_2_ and S_1_ states (S_x_ and 1B_u_^−^ states, for example) of carotene, carotenal, and other carotenoids have been reported^23,30,37^. This well represents very complex excited state dynamics of carotenoids.

Metal enhanced fluorescence (MEF) has been applied in many chemical and biological systems where the fluorescence signals are amplified with increased photostability^38–41^. Although MEF has been extensively investigated, the details of the MEF mechanism is not clearly understood yet. The increased local electric field near (<60 nm) the plasmonic nanoparticles amplifies both the absorption and the emission of the fluorophore, which is often called the electric field effect of MEF^40,42,43^. The decreases in the radiative lifetime of the fluorophore, which was first proposed as the induced plasmon effect of MEF, have recently been understood as the surface plasmon coupled emission (SPCE) or the fluorophore radiation through the scattering mode of the nanoparticles^39,44–46^.

Recently, we reported MEF of the laser dyes 4-(dicyanomethylene)-2-methyl-6-(4-dimethylaminostyryl)-4*H*-pyran (DCM) and Rhodamine 6 G dispersed in thin polymer films in the presence of the silver island film (SIF)^47^. The picosecond time-resolved fluorescence measurements probed the SPCE occurring with a much faster lifetime of ~400 ps than the original lifetimes of fluorophores (2.2–3.3 ns)^37^. The laser dye DCM also showed a strong MEF when a thin solution layer was directly located next to the SIF, where a much faster energy transfer (40–60 ps) from the silver nanoparticles to DCM molecules was observed by femtosecond transient absorption spectroscopy^48^.

In this paper, we extend our effort in the surface enhanced fluorescence by the silver or gold metal nanoparticles to natural photosynthetic pigments with very small fluorescence quantum yields. We have investigated the fluorescence enhancements of both carotenoids in the proximity of the silver substrate by time-resolved fluorescence measurements.

## Results and Discussion

### Metal-enhanced fluorescence of carotene and carotenal with the SIF

**Figure 2:**
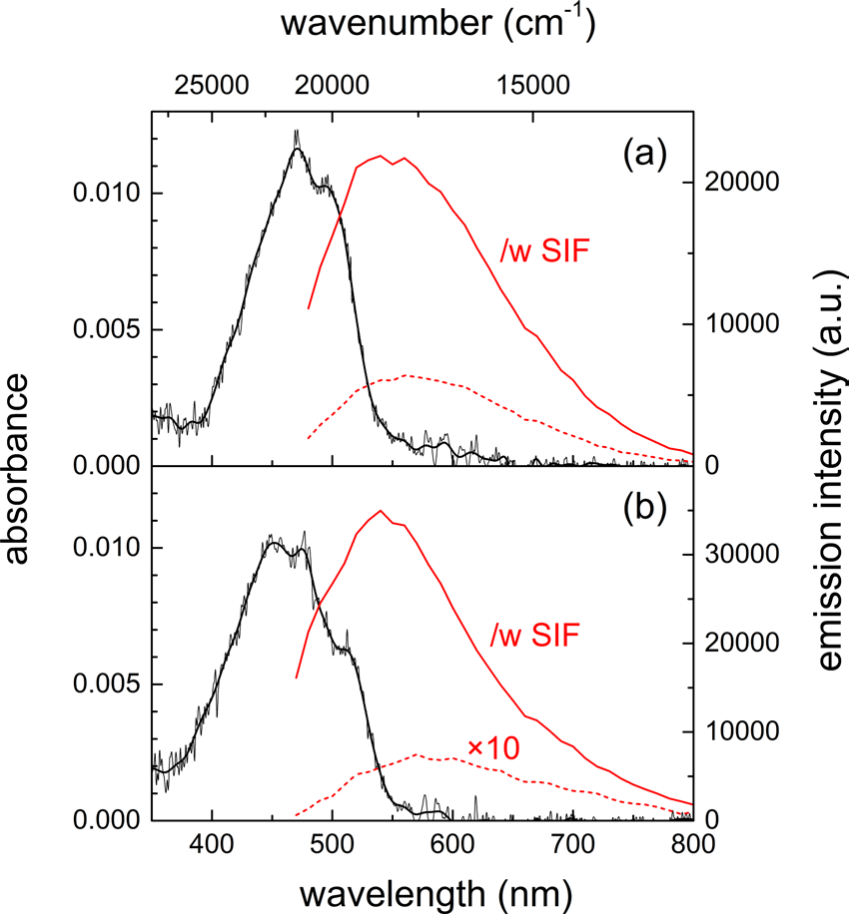
Absorption (black) and emission (red) spectra of all-*trans*-β-carotene (carotene) in (**a**) PS and (**b**) PEG film. Solid and dashed red lines represent the fluorescence spectra (λ_ex_ = 405 nm) of carotene with and without SIF, respectively. Emission spectrum of carotene without SIF in PEG was augmented by 10 times for comparison.

Figure 2 shows the steady-state absorption and emission spectra of carotene in thin polystyrene (PS) and polyethylene glycol (PEG) films and the main spectral features of both carotenoids in polymer films are summarized in Table 1. The absorption bands of carotene measured in the absence of the SIF appeared at 471 and 498 nm in a PS film and at 452, 475, and 514 nm in a PEG film. Carotenoids in the polymer films including PS and polymethylmethacrylate (PMMA) have been used in linear and nonlinear optical investigations, where the energy levels of the S_2_ state in the absorption spectrum were estimated from the polarizability index, *R*(ε) = (ε − 1)/(ε + 2) of the solvent/matrix^49–52^. Kwak *et al*. investigated the absorption and emission spectrum of a laser dye DCM in various polymer matrices, where the absorption and emission wavelengths and the emission lifetime of DCM were strongly dependent on the matrix polarity (dielectric constants ε = 2.5 and 14 were reported for PS and PEG polymer, respectively)^53^. The strong dependence on the matrix polarity reported for DCM is strongly related to the intramolecular charge transfer character of the S_1_ state of DCM, which is in general not applicable to the absorption spectra of carotenoids.

**Table 1:** Steady-state absorption and emission spectrum of carotene and carotenal in PS and PEG films.

Polymer	Absorption (nm)	Emission		
		Without SIF (nm)	With SIF (nm)	Enhancement
all-*trans*-β-carotene (**carotene**)				
PS	471, 498	568	550	3.4
PEG	452, 475, 514	585	545	47.3
8′-apo-β-caroten-8′-al (**carotena**l)				
PS	482	595	595	4.0
PEG	453, 496^sh^	595	585	9.8

^sh^shoulder band.

The absorption maximum of carotene (471 nm) in a PS film showed a good correlation with the polarizability dependence obtained from the solutions^25,54^. However, the absorption maximum of carotene in a PEG film appeared at 452 nm, which is blue-shifted from the absorption maximum of carotene in the less polar PS matrix. The absorption changes of the carotenoids in the polymer films with the introduction of the SIF cannot be measured due to the very small absorbance of carotenoids compared to that of the SIF.

The emission spectra of carotene were centered at 568 and 585 nm in the PS and PEG film, respectively, without the SIF. The emission bands of carotene in solutions were observed in the similar wavelength range of 524–571 nm with a linear relationship with the polarizability index of the solvent^25^. The emission spectra of carotene in both polymer films appear quite similar to each other with the introduction of the SIF while the emission with the PS film showed a blue-shift of 20 nm and the emission with the PEG film showed an opposite blue-shift of 40 nm. Since the emission intensities of carotene in the PS and PEG films were strongly increased by 3.4 and 47.3 times, respectively, with the introduction of the SIF, the common spectral properties of carotene with the SIF may be strongly related to those of the SPCE of the silver nanoparticles.

**Figure 3:**
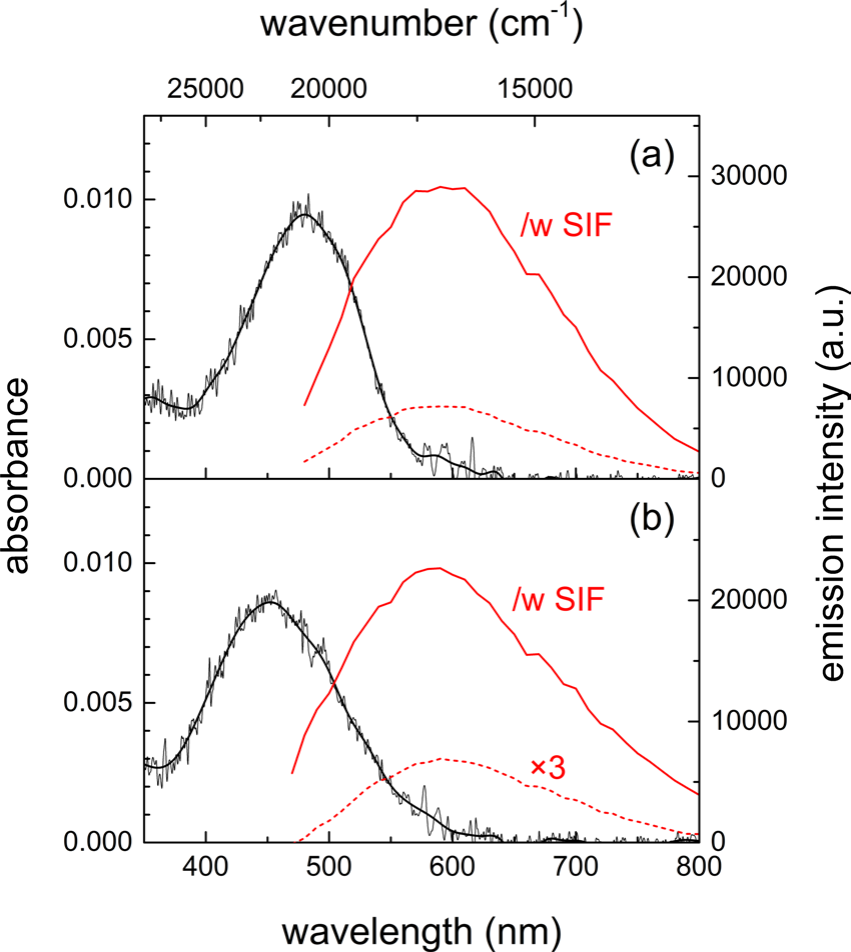
Absorption (black) and emission (red) spectra of 8′-apo-β-caroten-8′-al (carotenal) in (**a**) PS and (**b**) PEG film. Solid and dashed red lines represent the fluorescence spectra (λ_ex_ = 405 nm) of carotenal with and without a SIF, respectively. Emission spectrum of carotenal without a SIF in PEG was augmented by 3 times for comparison.

The absorption and emission spectra of carotenal in the PS and PEG films were shown in Fig. 3. The absorption bands of carotenal in the PS and PEG films appeared as broad bands centered at 482 and 453 nm, respectively, without the SIF. The absorption spectrum of carotenal in the solution phase showed a strong solvent dependence. The absorption spectrum of carotenal with the separated vibronic bands at ~435, 460, and 487 nm in cyclohexane (nonpolar) becomes a broad spectrum centered at 475 nm in chloroform (polar)^11^. The absorption band of carotenal in a PS film appeared at similar wavelength as the absorption band in chloroform solution. Whereas, the absorption band of carotenal in a PEG film appeared as blue-shifted from that in a PS film and chloroform solution, which is also observed for the carotene in a PEG film.

The blue-shifts of absorption bands in a PEG film observed from both carotenoids may not be explained with the polarizability index model where the dielectric constant of the PEG matrix is considered fairly high. The absorption band of a laser dye DCM showed small red-shifts upon the increase of ε in polymer matrices including PS and PEG^53^. The emission spectrum of DCM in the polymer matrices also showed strong red-shifts in polar matrix (560 → 603 nm), which is understood as the stabilization of the intramolecular charge transfer state of S_1_ in polar media^53^. However, the blue-shifts in the absorption bands of carotene and carotenal in the PEG matrix can be interpreted instead as the broken symmetry in the selection rules of molecules especially in a polar matrix. The oscillator strengths between the ground and the upper vibronic states of S_2_ may increase while those on to the lower vibronic states of S_2_ decrease in polar media. Then, these oscillator strength changes would appear as the blue-shifts of the S_0_ → S_2_ absorption bands.

The emission spectra of carotenal in the PS and PEG films appeared quite similar to each other without the SIF (both centered at 595 nm) and even with the introduction of the SIF (centered at 595 nm in the PS and 585 nm in the PEG matrices). The similar emission spectra of carotenal in both polymer matrices are similarly understood as the appearance of the SPCE with the introduction of SIF, where the emission intensities of carotenal in the PS and PEG film were increased by 4.0 and 9.8 times, respectively. The emission spectra of carotenal in solution also showed a strong dependence on the solvent polarity or polarizability. The emission bands at 550, 559, and 585 nm were observed from *n*-hexane, CCl_4_, and CS_2_ solutions, respectively, which originates from the S_2_ state^27,29^. The emission from the S_1_ state of carotenal was separately observed at 750 and 759 nm in CCl_4_ and CS_2_ solution, respectively^27^.

By considering the emission wavelengths only, the enhanced emission of both carotenoids in the PS and PEG films with the SIF may originate from the S_2_ state or the intermediate state between S_2_ and S_1_. The emission of carotene with the SIF appear at 545–550 nm (18,200–18,300 cm^−1^) and that of carotenal at 585–595 nm (16,800–17,100 cm^−1^). In addition, the long tails (700–800 nm) in the emission spectra of both carotenoids may also be considered as small contributions from the S_1_ emission. However, the emission lifetime of both carotenoids in the PS and PEG films are clearly different from the ultrafast (100–200 fs) dynamics of the S_2_ state, which will be clarified in the time-resolved absorption and emission measurements.

### Emission kinetics of both carotenoids in polymer films

**Figure 4:**
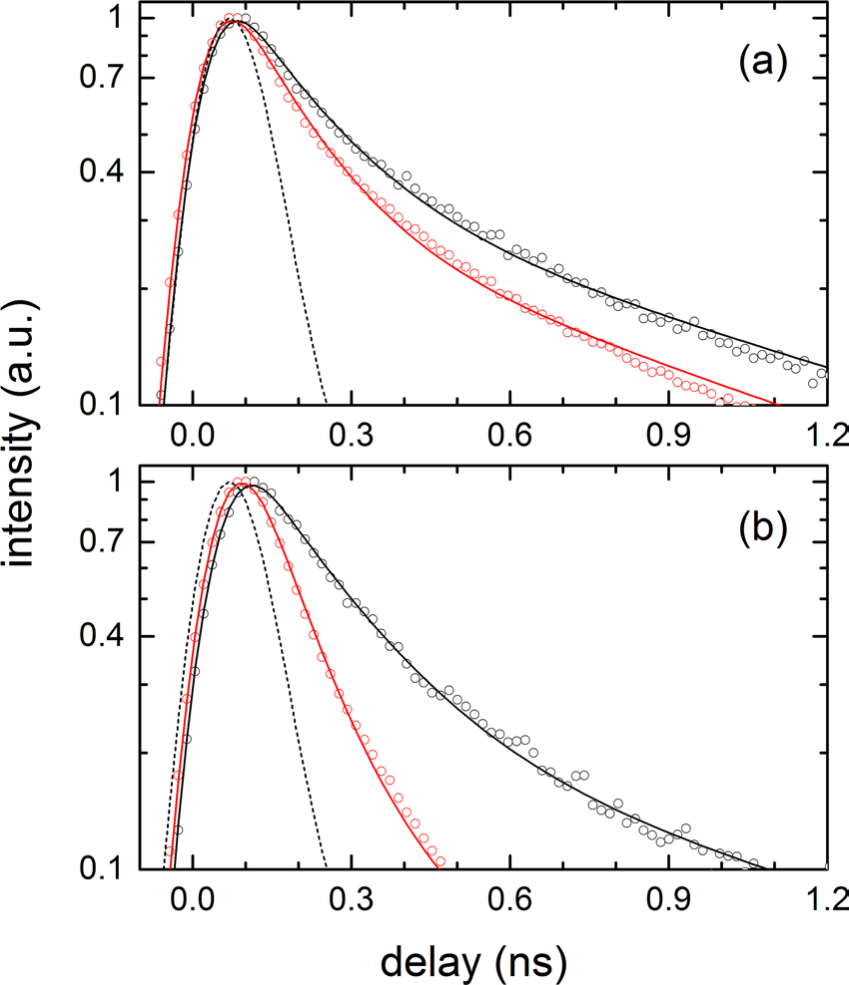
Emission kinetics (λ_ex_ = 405 nm) of all-*trans*-β-carotene (carotene) in (**a**) PS and (**b**) PEG film measured at 570–580 nm. Red circles (data) and lines (fit) represent the kinetics with the SIF, and black circles (data) and lines (fit) represent one without the SIF. Black dashed lines are the instrument response function (IRF; ~150 ps in FWHM) of time-resolved fluorescence measurements.

The emission kinetics of carotene in the PS and PEG films probed at 570–580 nm with and without the SIF are shown in Fig. 4. The small emission background signals from the polymer film or the SIF were separately measured and subtracted from the observed emission intensities of the carotenoid samples. The emission kinetics of carotene in polymer films were analysed with two exponential functions convoluted with the instrument response function (IRF). A faster (0.16–0.18 ns) and slower (1.1–1.2 ns) decay components were similarly found in the PS and PEG films without the SIF. With the introduction of the SIF, however, these lifetimes were further shortened and a clear difference between the carotene samples in the PS and PEG film appeared. The lifetimes of carotene were slightly shortened as 0.13 and 0.92 ns in the PS film, but much shortened as 0.09 and 0.56 ns in the PEG film. The strong enhancement (47.3 times) of emission in the carotene/PEG sample shown in Fig. 2(b) may originate from the shortened emission lifetimes of carotene, in other words, the increase of radiative rate constant.

**Figure 5:**
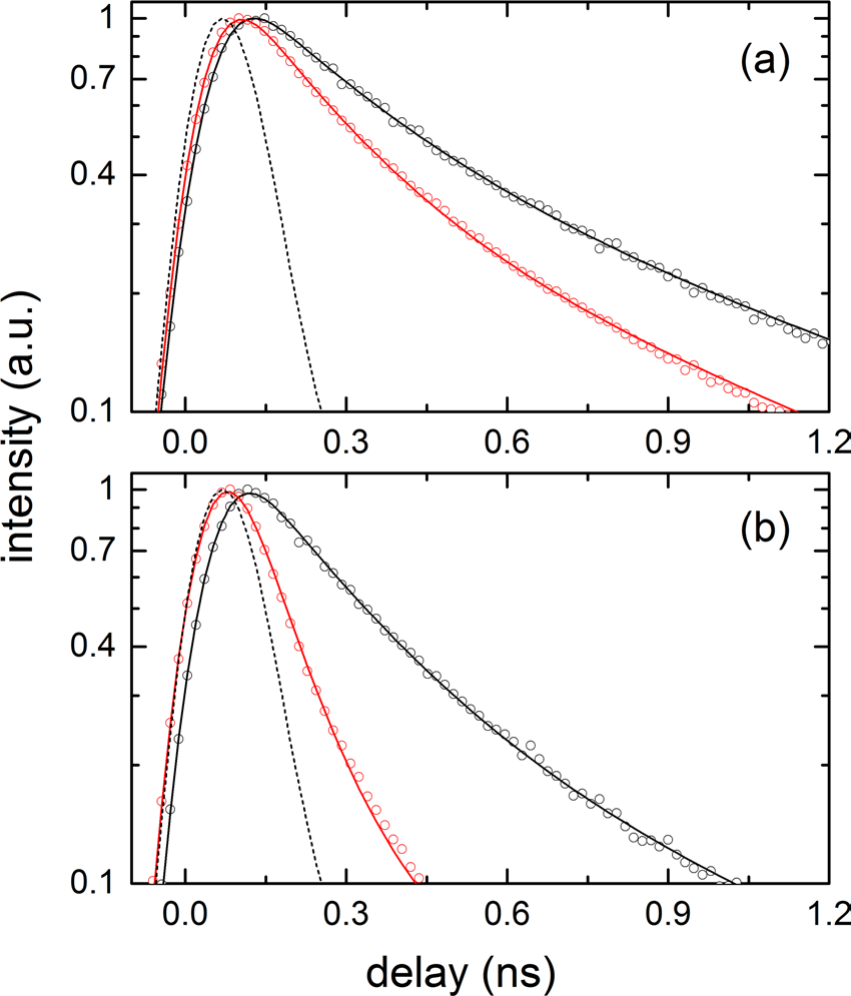
Emission kinetics (λ_ex_ = 405 nm) of 8′-apo-β-caroten-8′-al (carotenal) in (**a**) PS and (**b**) PEG film measured at 570–580 nm. Red circles (data) and lines (fit) represent the kinetics with the SIF, and black circles (data) and lines (fit) represent one without the SIF. Black dashed lines represent the IRF of time-resolved fluorescence measurements.

The emission kinetics of carotenal in both polymer films probed at 570–580 nm with and without the SIF are also shown in Fig. 5. Without the SIF, the decay lifetimes of carotenal showed no significant difference between two polymer films; 0.20–0.24 ns and 0.85–0.94 ns. The emission lifetimes of carotenal became further shortened with the SIF, and the decrease of the lifetime was much greater with the PEG film. The lifetimes of 0.18 and 0.80 ns for the PS film and 0.09 and 0.49 ns for the PEG film were resolved. The emission lifetimes of carotenal were sharply decreased in the PEG film with the SIF, where a larger emission enhancement (9.8 times) was observed compared to 4.0 times in the PS film as shown in Fig. 3(b). The details of the emission kinetics of both carotenoids are summarized in Table 2.

**Table 2:** Exponential fit parameters for the emission kinetics of both carotenoids with 405 nm excitation probed at 575 nm.

Polymer	Without SIF		With SIF		*k*_ET_ (s^−1^, efficiency)	
	τ_1_ (ns)	τ_2_ (ns)	τ_1_ (ns)	τ_2_ (ns)	1^st^ interm.	2^nd^ interm.
**all-trans-β-carotene (carotene)**						
PS	0.16 ± 0.00 (0.75)^a^	1.07 ± 0.01 (0.25)	0.13 ± 0.00 (0.80)	0.92 ± 0.01 (0.20)	1.4 × 10^9^ (19%)	1.5 × 10^8^ (14%)
PEG	0.18 ± 0.00 (0.84)	1.19 ± 0.02 (0.16)	0.09 ± 0.00 (0.92)	0.56 ± 0.01 (0.08)	5.6 × 10^9^ (50%)	9.5 × 10^8^ (53%)
**8′-apo-β-caroten-8′-al (carotenal)**						
PS	0.24 ± 0.00 (0.65)	0.94 ± 0.01 (0.35)	0.18 ± 0.00 (0.75)	0.80 ± 0.01 (0.25)	1.4 × 10^9^ (25%)	1.9 × 10^8^ (15%)
PEG	0.20 ± 0.00 (0.80)	0.85 ± 0.01 (0.20)	0.09 ± 0.00 (0.92)	0.49 ± 0.01 (0.08)	6.1 × 10^9^ (55%)	8.6 × 10^8^ (42%)

^a^Numbers inside the parentheses denote the ratio of amplitude.

**Figure 6:**
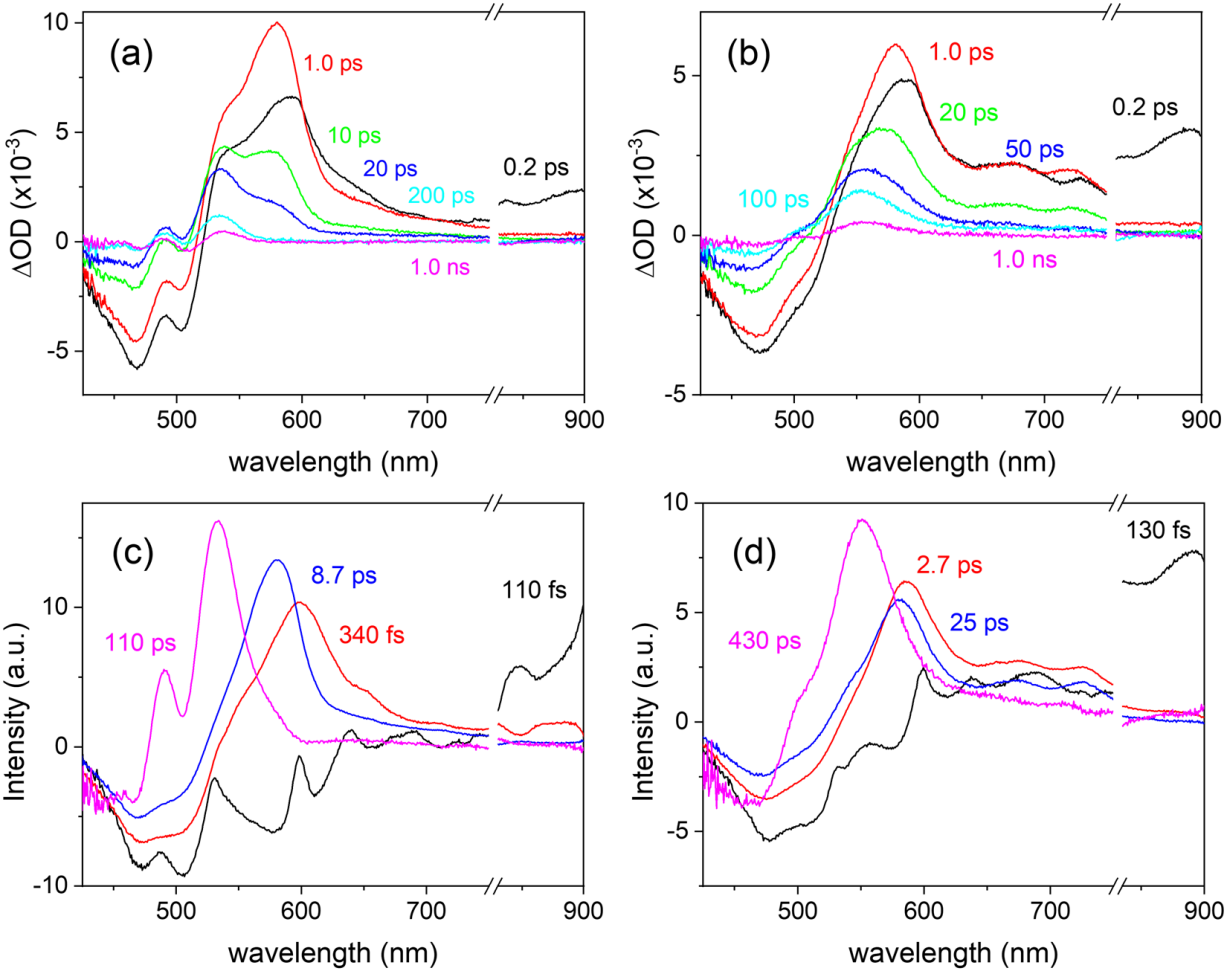
Femtosecond transient absorption spectra (λ_ex_ = 403 nm) of (**a**) carotene and (**b**) carotenal in PS films. The species associated difference spectra (SADS) of (**c**) carotene and (**d**) carotenal obtained from the global analysis of the transient absorption results.

The excited-state dynamics of carotenoids in polymer films have not been reported to the best of our knowledge, but the ultrafast dynamics of carotenoids in protein environments such as the photosynthetic complexes have been numerously reported^7^. Thus the S_2_ dynamics of both carotenoids in the polymer matrices can also be assumed as occurring in ultrafast time scales (100–200 fs) and the precise measurement of the excited state lifetimes of carotenoids in polymer films is not possible due to the time resolution (~150 ps) of TCSPC measurements. Thus we performed femtosecond transient absorption measurements on the carotene and carotenal in the PS film, and the results are summarized in Fig. 6. Unfortunately, transient absorption measurements on the PEG film samples and any sample with the SIF were not possible due to the significant photodamage from the femtosecond pump pulses. The excited state dynamics of the S_2_ and S_1_ states relevant to those in the solution phase were observed including the excited state absorption of the hot S_1_ state (often claimed as the intermediate state between the S_2_ and S_1_ states). The global analysis with a branching model from the *bright* S_2_ state (S_2_ → hot S_1_ → S_1_ → S_0_ and S_2_ → *intermediate* states → S_0_) well supported the transient absorption results, and the branching ratio of 4:1 was found from the relative ratio between the transient absorption signals from the S_1_ and the intermediate states of both carotenoids. The species associated difference spectra (SADS) representing the S_2_ state (110 and 130 fs for carotene and carontenal, respectively), the hot S_1_ state (340 fs and 2.7 ps), the S_1_ state (8.7 and 25 ps) are shown in Fig. 6(c,d). It is interesting to note that a separate state directly populated from the S_2_ state was found in both carotenoids, and the lifetimes of the intermediate states (110 and 430 ps for carotene and carotenal, respectively) are similar to the faster components in the TCSPC measurements (0.16 and 0.24 ns for carotene and carotenal, respectively). The 430 ps lifetime of carotenal retrieved from the global analysis of the transient absorption data seems to be longer than the 0.24 ns lifetime from TCSPC measurements. However, the transient absorption data between the time window of 0–1.2 ns may not be enough for distinguishing two emission kinetics of similar lifetimes (0.24 and 0.94 ns, for example). Thus all the kinetic information obtained from TCSPC measurements are considered as accurate and will be used in the further analysis.

**Figure 7:**
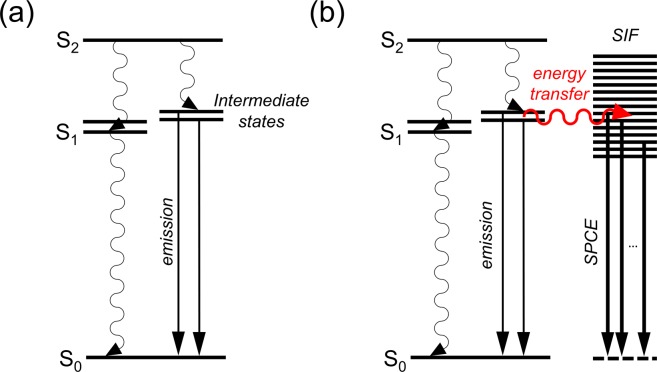
The energy level diagrams of carotenoids in the polymer matrices (**a**) without and (**b**) with the presence of the SIF.

From the transient absorption measurements, we found that the emission of both carotenoids in the PS and PEG films originates from the intermediate states between the S_2_ and the S_1_ state, not from the S_2_ or S_1_ state. The shorter kinetic components (0.16 ns for carotene and 0.24 ns for carotenal) from the TA measurements and the longer components (0.9 ns for carotene and 1.1 ns for carotenal) from the TCSPC measurements clearly show a bifurcated dynamics for the intermediate states of both carotenoids. This clearly shows the possibility for the presence of multiple intermediate states in the polymer matrices. It is reported that the intermediate states of carotenoids between the S_2_ and S_1_ states may be related to the structural conformation changes in the polyene backbones^10,37,55^. We propose that the strongly enhanced emission of both carotenoids in the polymer matrices may be related to the structural deformation of carotenoids. Since the emission of both carotenoids exhibited a significant amount of emission in the longer wavelength of 700–800 nm, the intermediate emitting state of both carotenoids located below the S_1_ state can also be considered. The detailed energy diagrams of both carotenoids in the polymer matrices without and with the presence of the SIFs are shown in Fig. 7.

### Mechanism of metal-enhanced fluorescence

In the PS films, both carotenoids showed relatively small (3.4 and 4.0 times) emission enhancements with the SIF. In addition, the emission kinetics of both carotenoids were slightly shortened in the PS films with the introduction of the SIF; 0.16 → 0.13 ns and 1.07 → 0.92 ns for carotene, and 0.24 → 0.18 ns and 0.94 → 0.80 ns for carotenal. On the other hand, emission enhancements of both carotenoids in the PEG film were much larger (47.3 and 9.8 times) and the emission lifetimes reduced strongly upon the introduction of the SIF; 0.18 → 0.09 ns and 1.19 → 0.56 ns for carotene, and 0.20 → 0.09 ns and 0.85 → 0.49 ns for carotenal.

The strong increases in the emission intensity and the reduction of fluorophore’s excited state lifetimes are considered as well-known evidence of so-called induced plasmon effect or SPCE, where the energy transfer from carotenoids to the surface plasmon of the silver nanoparticles is known to occur^39–41^. From the emission lifetimes of both carotenoids observed in the polymer films, the rate constants for the energy transfer from the 1^st^ and 2^nd^ intermediate states of carotenoids and energy transfer efficiencies were determined, and summarized in Table 2. In the PS film, the rate constant for the energy transfer, *k*_ET_ = 1.4 × 10^9^ s^−1^ (for both carotenoids) and energy transfer efficiency of 19% (carotene) and 25% (carotenal) were determined from the 1^st^ intermediate emission kinetics. From the 2^nd^ intermediate emission kinetics, *k*_ET_ = 1.5 × 10^8^ s^−1^ (carotene) and 1.9 × 10^8^ s^−1^ (carotenal), and the efficiency of 14% (carotene) and 15% (carotenal) were obtained. In the same manner, *k*_ET_ = 5.6 × 10^9^ s^−1^ (carotene) and 6.1 × 10^9^ s^−1^ (carotenal), and the efficiency of 50% (carotene) and 55% (carotenal) were determined from the 1^st^ intermediate emission kinetics in the PEG film. From the 2^nd^ intermediate emission kinetics in the PEG film, *k*_ET_ = 9.5 × 10^8^ s^−1^ (carotene) and 8.6 × 10^8^ s^−1^ (carotenal), and the efficiency of 53% (carotene) and 42% (carotenal) were determined. The efficient energy transfer (40–55%) from both the intermediate states of carotenoids was observed from the PEG matrices, which is closely related to the strong enhancement (47.3 and 9.8 times) of the emission spectrum of both carotenoids.

Due to very weak absorbance (~0.01) of carotenoids dispersed in polymer films, the amount of the extinction enhancements by the introduction of the SIF were not determined in our experiments. A small increase in the emission intensity originating from the increased excitation of carotenoids is possible, which is also be strongly dependent on the distance between the molecules and the metal nanoparticles. Nonetheless, the strong emission enhancements of carotenoids in the PS and PEG films can be considered as mainly originating from the partial energy transfer from the intermediate states of carotenoids to the surface plasmons and the subsequent emission from the silver nanosurfaces.

It is interesting to note that carotenoids dispersed in the PEG films showed larger fluorescence enhancements and greater decreases in the intermediate state lifetimes (thus more efficient energy transfer to the surface plasmons of nanoparticles) with the SIF. Especially, carotene showed a fairly large (47.3 times) emission enhancement with the SIF. The strong emission enhancements and efficient energy transfer to nanoparticles are considered as strongly related to the changes in the absorption and emission spectra of carotenoids in the polymer films. First of all, the emission spectra of carotenoids showed strong blue-shifts (585 → 545 nm for carotene in PEG) when the emission intensity is greatly enhanced by the introduction of the SIF, as shown in Fig. 2(b). In other words, the emission spectra of carotenoids which are strongly enhanced by the SIF are clearly different from ones obtained without the SIF. This is considered as one of evidences for the SPCE initiated by the energy transfer from carotenoids to nanoparticles, where the emission spectra of the surface plasmon may be strongly coupled to the scattering portion of the extinction spectrum of the SIF^48,56^ and thus blue-shifted compared to the emission spectra without the SIF. In addition, the absorption spectra of carotenoids in the PEG matrices are slightly blue-shifted (20–30 nm) from the absorption bands in the PS matrices, which may also be considered as related to the efficient energy transfer from the intermediate excited states to the surface plasmons of silver nanoparticles.

### Statistical analysis of surface homogeneity

**Figure 8:**
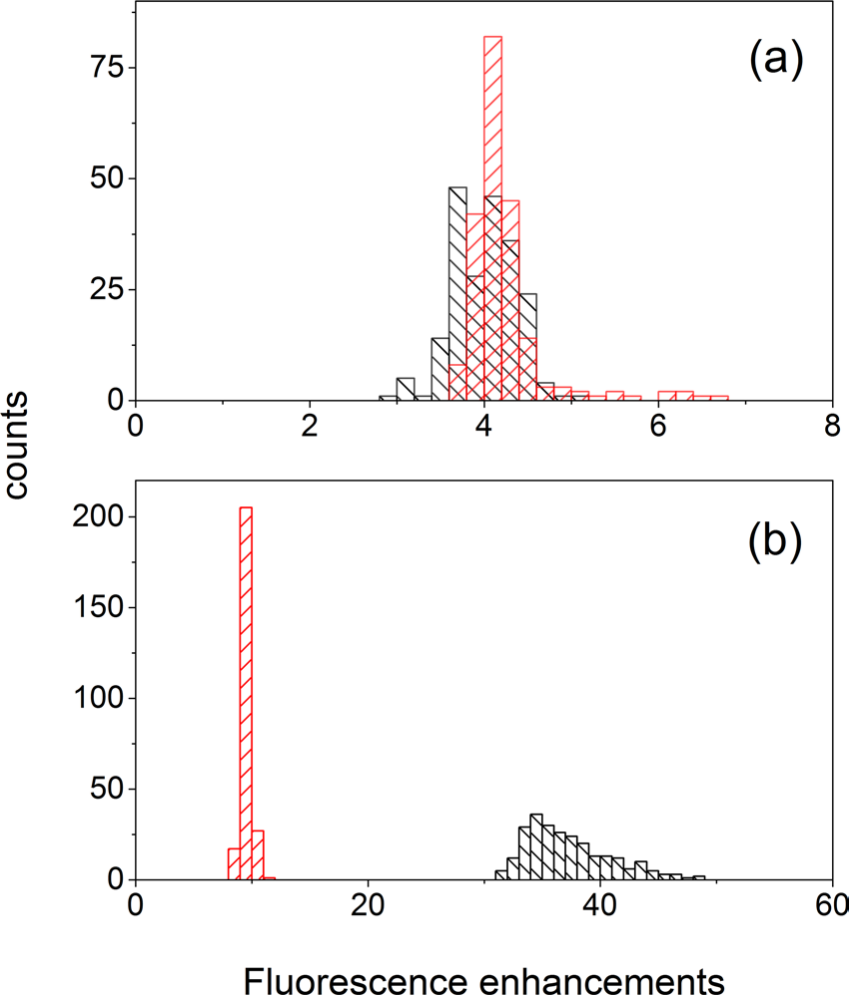
Histograms for the fluorescence intensities of carotene in (**a**) PS and (**b**) PEG film, and of carotenal in (**c**) PS and (**d**) PEG film. The black reprensents the result without the SIF, and the red the results with the SIF. Fluorescence intensities were measured at the peak position of each spectrum.

To reveal further details of the emission enhancements of carotenoids with the SIF and possible effects from the local hot spots in the silver surface, statistical measurements on the emission enhancement of both carotenoids were performed. By translating the sample slowly, the emission measurements from about 250 random spots in the sample area were measured. The statistical observations were plotted as histograms in Fig. 8. The histograms for the emission of both carotenoids without the SIF showed narrow distributions of the emission intensity. This represents that the carotenoid molecules were evenly distributed in the PS and PEG films and that the emission quantum yield of each carotenoid molecule showed quite a narrow distribution. However, the histograms for the emission signals of both carotenoids in the PS and PEG films with the SIF appeared as much broadened, mostly homogeneously. This represents the size or shape distribution of the silver island of the SIFs used in the MEF, which may be unavoidable in the inhomogeneous SIFs.

**Figure 9:**
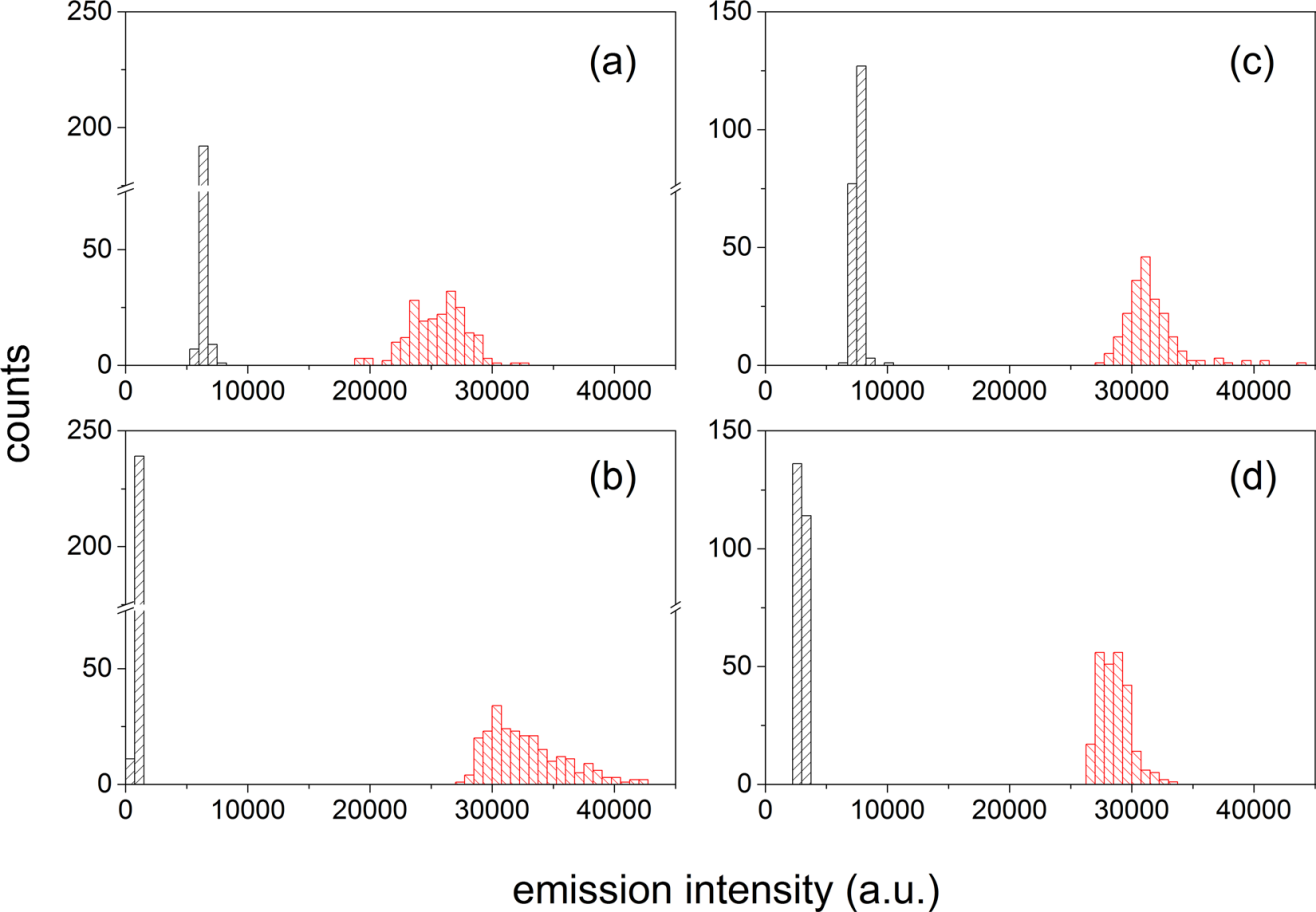
Histograms for the fluorescence enhancements of carotene (black) and carotenal (red) in (**a**) PS and (**b**) PEG films.

The distributions in the emission enhancements for both carotenoids in the polymer films were then visualized in the histograms for the emission enhancement factor as shown in Fig. 9. In the PS film, both carotenoids showed a similar 4 times emission enhancement with the SIFs. However, clear differences between two carotenoid samples were shown from the results with the PEG film. Carotenal showed a narrow distribution of emission enhancements with the average 10 times enhancements while carotene displayed a much broader distribution with the average emission enhancement of 38 times. Although similarly efficient energy transfer of 50–55% was observed for carotene and carotenal from the emission dynamics measurements, the emission enhancement of carotene and carotenal showed a clear difference in the dependence on the local nanostructure of the silver islands. This would mean that the emission enhancements of both carotenoids in the PS and PEG films may occur in multiple ways including the electric field effect and SPCE. In other words, the emission enhancements of 47 and 10 times observed from the carotene and carotenal in the PEG film cannot be solely attributed to the SPCE. In order to quantify the emission enhancements from the increase of the local electric filed and from the SPCE initiated from the energy transfer from the fluorophore to nanoparticles, further experimental efforts are essential.

The surface plasmon properties of metal nanoparticles are strongly dependent on the shape and size of nanoparticles, thus a further exploration of fluorescence enhancements on more homogeneous silver surfaces might be needed to reveal the details of MEF. Silver colloidal surfaces composed of highly monodisperse spherical nanoparticles of 20–200 nm in diameter which are synthesized by a kinetic-controlled seeded-growth method will be a good testbed of MEF of dyes including carotenoids^57,58^.

In conclusion, the strong enhancements of carotenoid emission which is inherently very weak have been shown from all-*trans*-β-carotene (carotene) and 8′-apo-β-caroten-8′-al (carotenal) in the PS and PEG matrices with the silver island films (SIFs). The emission intensity and dynamics measurements by a TCSPC setup reveals that the emission enhancements mainly originate from the energy transfer from the carotenoid excited states to the surface plasmons and the subsequent plasmon coupled emission (SPCE). The energy transfer efficiencies from the S_2_/S_1_ intermediate states were determined as 19–25% for the PS and 50–55% for the PEG matrices, which seems to be strongly related to the relative energy levels of the excited states of carotenoids in the polymer films. Further experimental efforts on a homogeneous metal nanosurface, for example, are needed to distinguish the fluorescence enhancements by the local field increases from ones by the energy transfer and subsequent SPCE.

## Methods

**Figure 10:**
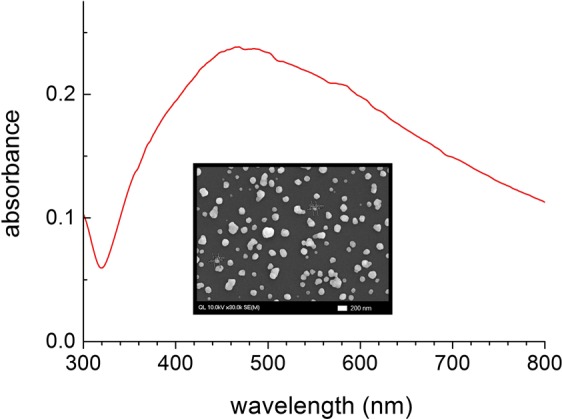
The extinction spectrum and a SEM image (inset) of the silver island film (SIF).

All-*trans*-β-carotene (Sigma-Aldrich, St. Louis, MO, USA), 8′-apo-β-caroten-8′-al (Santa Cruz Biotechnology, Dallas, TX, USA) were purified by high performance liquid chromatography, and all other chemicals were used without further purification. The SIFs with the absorption maximum of 0.2–0.3 around 400–550 nm were synthesized by Tollen’s method^59^, which we found optimal for the emission enhancements with many dye molecules. The extinction spectrum and the SEM image of the SIFs used in this work are shown in Fig. 10. The SEM measurements by a FE-SEM (Hitachi 4700) revealed the average diameter of 80–170 nm for the silver islands^48^. The half of silver substrate was removed by a dilute nitric acid solution for the fluorescence enhancement measurements.

The 1.0% (w/v) solutions of PS (avg. m.w. 208,000; Wako Pure Chemical, Osaka, Japan) and PEG (avg. m.w. 1,850–2,150; Samchun Pure Chemical, Seoul, Korea) in THF which contain carotenoids were spin-coated on the SIF substrates and the thickness of the films was measured as <100 nm. The concentration of carotenoids in the polymer solutions was set as 0.2 mM and the fused silica substrates (Spectrosil 200, UQG Optics, Cambridge, England) with a minimal fluorescence background signal were used for all the fluorescence measurements.

Time-resolved fluorescence spectra have been measured by a time-correlated single photon counting (TCSPC) module (PicoHarp 300, PicoQuant) with a picosecond pulsed laser (P-C-405; PicoQuant)^47,60^. A *f* = 260 mm monochromator (Cornerstone 260, Newport) and a photomultiplier tube detector (PMA 192, PicoQuant) were used to measure fluorescence signals with a ~5 nm spectral resolution. The pulse energy of 10 pJ (at 10 MHz) was used, and the film samples were moved linearly at a speed of 0.25 mm/sec for all spectral and kinetic measurements in order to minimize photodamage from laser excitation. The excitation pulses were defocused to a diameter of 150 μm at the sample to minimize any photodamage and to measure the emission signals from a wider area of the samples. Thus, the observed emission signals with and without the SIF would not be directly related to the inhomogeneity of the silver islands. Repeated absorption and emission measurements (with and without the SIF) at random sample positions were also performed to avoid any effect from the sample inhomogeneity. The IRF of TCSPC set-up was measured as ~150 ps by using a fused-silica window at the sample position and considered as compatible with the previously reported values^61^.

A home build transient absorption spectrometer was used to measure the excited state dynamics of both carotenoids in thin polymer films. The details of the transient absorption measurements were described elsewhere^48,62^.

## Data Availability

The datasets generated during and/or analyzed during current study are available from the corresponding author on reasonable request.
